# Amyloid A amyloidosis with subcutaneous drug abuse

**DOI:** 10.12861/jrip.2014.06

**Published:** 2013-11-02

**Authors:** Jair Munoz Mendoza, Vasil Peev, Mario A Ponce, David B Thomas, Ali Nayer

**Affiliations:** ^1^Division of Nephrology and Hypertension, University of Miami, USA; ^2^Department of Medicine, University of Miami, USA; ^3^Renal Pathology Laboratory, University of Miami, USA

**Keywords:** AA amyloidosis, Suppurative skin infections, Subcutaneous drug abuse, Proteinuria, Renal failure

## Abstract

**Introduction:** Amyloid A (AA) amyloidosis is a systemic form of amyloidosis secondary to chronic infections and inflammatory disorders. An acute-phase protein produced by the liver, serum amyloid A (SAA) is the precursor of AA amyloid fibrils. AA amyloid deposition occurs predominantly in the kidneys, spleen, adrenal glands, liver and gastrointestinal tract. The manifestations of AA amyloidosis involving the kidneys include proteinuria, tubular dysfunction and progressive loss of renal function.

**Case:** We report a 47-year-old drug addict who developed AA amyloidosis as a result of recurrent suppurative skin infections secondary to subcutaneous drug injection. Elevated C-reactive protein concentrations attested to the presence of a chronic systemic inflammatory state. He suffered from the nephrotic syndrome and insidious loss of renal function. Isosthenuria and glycosuria were indicative of renal tubular dysfunction. Renal biopsy demonstrated AA amyloidosis involving the glomeruli, tubular basement membranes and blood vessel walls.

**Conclusion:** Superimposed acute tubular necrosis due to concomitant endocarditis and cocaine use accelerated his renal disease. Case presentation is followed by a brief discussion of clinical features, natural history and outcome of AA amyloidosis with a particular emphasis on AA amyloidosis as a complication of subcutaneous drug abuse.

**Implication for health policy/practice/research/medical education:**

Amyloid A (AA) amyloidosis is a systemic form of amyloidosis secondary to chronic infections and inflammatory disorders. An acute-phase protein produced by the liver, serum amyloid A (SAA) is the precursor of AA amyloid fibrils. We report a 47-year-old drug addict who developed AA amyloidosis as a result of recurrent suppurative skin infections secondary to subcutaneous drug injection. Renal biopsy demonstrated AA amyloidosis involving the glomeruli, tubular basement membranes and blood vessel walls. Superimposed acute tubular necrosis due to concomitant endocarditis and cocaine use accelerated his renal disease. Case presentation is followed by a brief discussion of clinical features, natural history and outcome of AA amyloidosis with a particular emphasis on AA amyloidosis as a complication of subcutaneous drug abuse.

## Case Presentation

A 47-year-old Caucasian man presented with a right arm abscess as a consequence of subcutaneous injection of narcotics. He was found to have proteinuria and renal insufficiency. The past medical history was notable for subcutaneous injection of heroin and cocaine complicated by skin abscesses in the upper and lower extremities over the past five years. In addition, he had hepatitis C infection diagnosed in 1998 and psoriasis. He never received treatment for hepatitis C. Review of systems was negative for fevers, chills, chest pain, shortness of breath, hematuria, dysuria, headaches, or blurry vision. He was homeless and snorted cocaine for 25 years.

**Figure 1:**
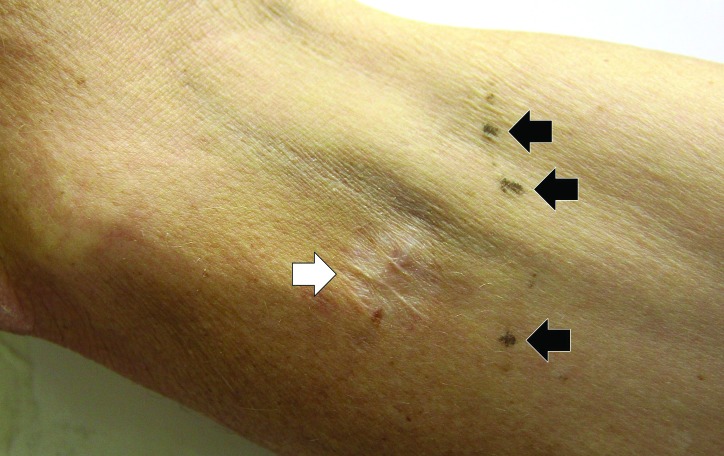
Cutaneous scar and needle marks.A localized cutaneous scar (white arrow) and several needle marks (black arrows) are seen on the volar aspect of the left arm.

On physical examination, the patient was a malnourished middle-aged man in no acute distress. The temperature was 37.0 °C, the blood pressure 157/89 mm Hg, the pulse 80 beats per minute, the respiratory rate 12 breaths per minute, the oxygen saturation 100% while breathing ambient air and the body mass index 19.8 kg/m^2^. Examination of upper and lower extremities demonstrated multiple needle marks, several localized cutaneous scars, and hyperkeratotic plaques on the elbows (Figure 1). A 2x3 cm abscess with purulent secretion and surrounding erythema was present on his right arm. The heart had a regular rate and rhythm. There was a 3/6 aortic diastolic murmur. The liver span was 20 cm. No splenomegaly was appreciated. There was 2+ pitting edema over shins.

Laboratory data are summarized in Table 1. Leukocytosis, neutrophilia, microcytic anemia and hypoalbuminemia were noted. Serum creatinine was 3.8 mg/dL and urea nitrogen concentration was 37 mg/dL on admission. Serum creatinine was 2.3 mg/dL four months prior to the presentation. Urinalysis revealed dilute urine positive for glucose and protein. Urinary protein excretion was estimated 7.3 g/day based on a random urine protein-to-creatinine ratio. A 24-hour urine collection revealed 5.4 g of protein. Serum protein electrophoresis showed no monoclonal bands, but increased acute-phase reactants were noted. Urine protein electrophoresis was negative for Bence-Jones proteins. Serologic tests revealed antibodies against hepatitis C and hepatitis B surface antigen. Hepatitis B surface antigen and antibodies against hepatitis B core antigen and HIV were not detected. Urine toxicology screen was positive for cocaine. C-reactive protein (CRP) concentration was 2.3 mg/dL. Culture from the skin abscess was negative and multiple blood cultures were negative for common bacteria and HACEK group (Hemophilus, Actinella, Cardiobacterium, Eikenella, Kingella). Renal ultrasonography revealed enlarged kidneys (left: 13 cm, right: 15 cm). There was no hydronephrosis, masses, or stones. Chest radiography was unremarkable. Abdominal Ultrasound confirmed hepatomegaly. Transthoracic echocardiography showed vegetations involving aortic and mitral valves. Transesophageal echocardiography showed 1.7 cm vegetation involving aortic valve causing severe aortic insufficiency and a small vegetation on the anterior mitral leaflet. The patient was started on vancomycin and piperacillin/tazobactam for skin abscess and culture-negative endocarditis.

**Table 1:** Laboratory data

**Analyte**	**On Admission**	**Reference Range**	**Analyte**	**On Admission**	**Reference Range**
Sodium (mmol/L)	137	135–145"	Urinalysis		
Potassium (mmol/L)	4.7	3.4–4.8	Color	Light yellow	Yellow
Chloride (mmol/L)	106	99–109	Appearance	Clear	Clear
Carbon dioxide (mmol/L)	**20**	21–30	pH	6.5	4.6–7.8
Urea nitrogen (mg/dL)	**37**	7–22	Specific gravity	1.006	1.001–1.035
Creatinine (mg/dL)	**3.8**	0.5–1.4	Glucose (mg/dL)	**30**	Negative
Glucose (mg/dL)	78	65–99	Blood	Negative	Negative
Calcium (mg/dL)	**8.4**	8.6–10.3	Protein (mg/dL)	**300**	Negative
Protein (g/dL)	**4.7**	6.3-8.2	White blood cells/hpf	0	0-2
Albumin (g/dL)	**2.1**	3.5–5.5	Red blood cells/hpf	2	0-2
Aspartate transaminase (IU/L)	26	15-46	Urine protein:creatinine	**7.3**	0.02-0.13
Alanine transaminase (IU/L)	28	9-52	UPEP	No monoclonal	No monoclonal
Alkaline phosphatas e (IU/L)	75	38-126	SPEP	No monoclonal	No monoclonal
Bilirubin, total (mg/dL)	0.2	0.3-1.3	CRP (mg/dL)	**2.3**	<1.0
Creatine phosphokinase (IU/L)	<20	38-234	Antinuclear antibodies	Negative	Negative
Lactate dehydrogenase (IU/L)	**456**	84-246	C3 (mg/dL)	124	90-180
Haptoglobin (mg/dL)	**237**	30-200	C4 (mg/dL)	**47**	10-40
Hemoglobin (g/dL)	**12.4**	13.6–16.7	HIV 1/2	Non-reactive	Non-reactive
White-cell count (x10^3^/mm^3^)	**12.0**	4.8–10.8	Hepatitis C Ab	**Positive**	Negative
Neutrophils (x10^3^/mm^3^)	**9.4**	1.5-7.0	Hepatitis C viral load	**53,900**	Negative
Platelet count (x10^3^/mm^3^)	**410**	130–350	Rheumatoid factor (IU/mL)	<10	<10
Urine toxicology screen	**Cocaine**	Negative	Cryoglobulins	Negative	Negative
APTT (sec)	31.3	24.5-35.7	Total cholesterol (mg/dL)	**249**	150-200
PT (sec)	11.3	10.1-12.6	Triglycerides (mg/dL)	**212**	40-160
Values out of the reference range are in bold. Ab, antibody; APTT, activated partial thromboplastin time; CRP, C-reactive protein; HIV, human immunodeficiency virus; PT, prothrombin time; SPEP, serum protein electrophoresis; UPEP, urine protein electrophoresis.					

To evaluate renal insufficiency and the nephrotic syndrome, renal biopsy was performed. There was accumulation of weakly PAS reactive, amorphous, and acellular material in glomerular mesangium, tubular basement membranes and blood vessel walls (Figure 2A,B). Polarizing microscopy of Congo red-stained tissue sections revealed apple-green birefringence of deposits (Figure 2C,D). Immunohistochemistry using antisera specific for serum amyloid A demonstrated strong staining of deposits (Figure 2E). In addition, severe acute tubular epithelial cell injury and scattered granular casts in tubular lumina were noted. There was moderate interstitial fibrosis and tubular atrophy. Moderate interstitial inflammation composed of mononuclear inflammatory cells and infrequent polymorphonuclear inflammatory cells was present. There was moderate arteriosclerosis and mild arteriolosclerosis. Immunofluorescence microscopy demonstrated weak staining for IgM and C1q in mesangial areas and segments of glomerular capillary walls. No kappa or lambda light chain deposition was detected. Ultrastructural examination revealed fine, non-branching, fibrillary material in the expanded mesangium and glomerular basement membranes (Figure 2F). A diagnosis of AA amyloidosis and acute tubular necrosis was rendered.

Figure 2. 
Renal AA amyloidosis. Accumulation of amorphous material in the glomerular mesangial areas (white arrows), tubular basement membranes (black arrow) and blood vessel walls (arrowhead) (A). Accumulation of amorphous material in the tubular basement membranes (white arrows) (B). Polarizing microscopy of a Congo red-stained tissue section revealed apple-green birefringence of deposits in the glomerular mesangial areas (white arrows) and tubular basement membranes (black arrows) (C,D). Immunohistochemistry using antisera specific for serum amyloid A demonstrating strong staining of deposits in glomeruli (E). Ultrastructural examination revealing fine non-branching fibrillary material in the expanded glomerular basement membrane (GBM) (F). Tissue sections stained with Masson’s trichrome (A), periodic acid-Schiff (B), Congo red (C) and anti-sera against serum amyloid A (E) were examined with a light microscope (A-C,E), an immunofluorescence microscope (D) and an electron microscope (F).

**A:**
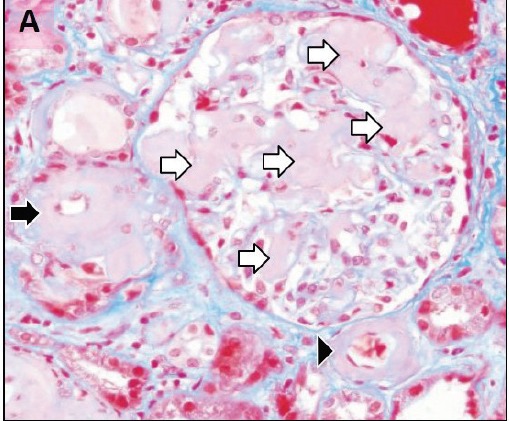

**B:**
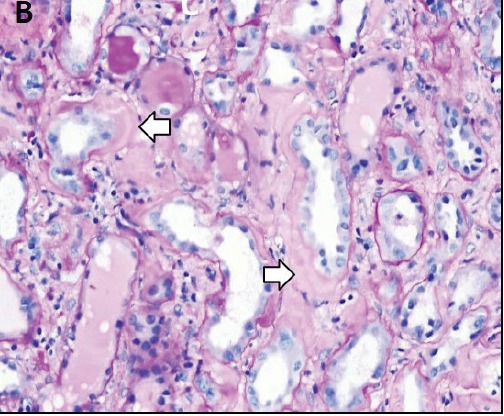

**C:**
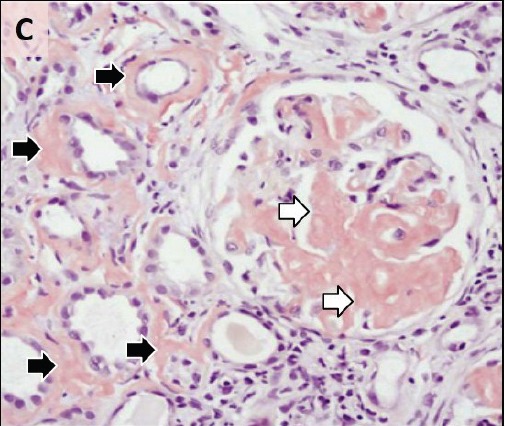

**D:**
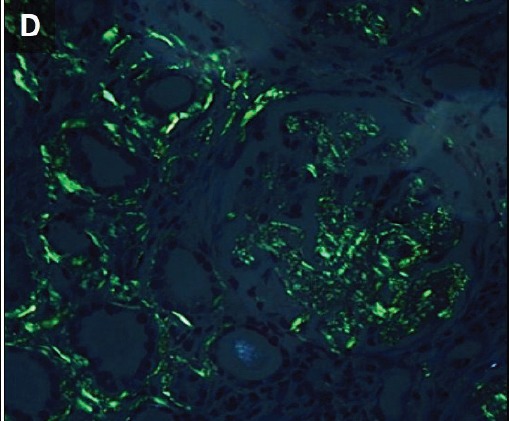

**E:**
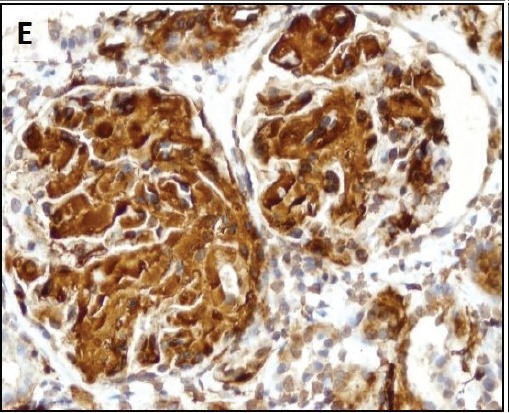

**F:**
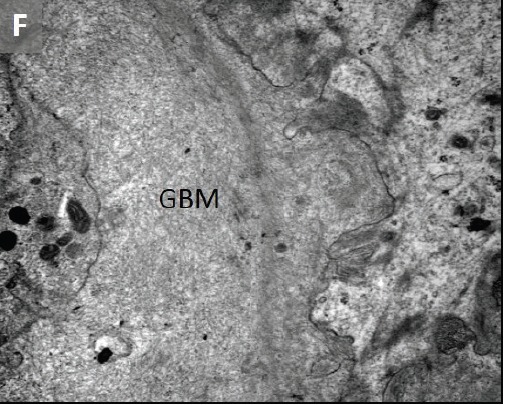

**Figure 3:**
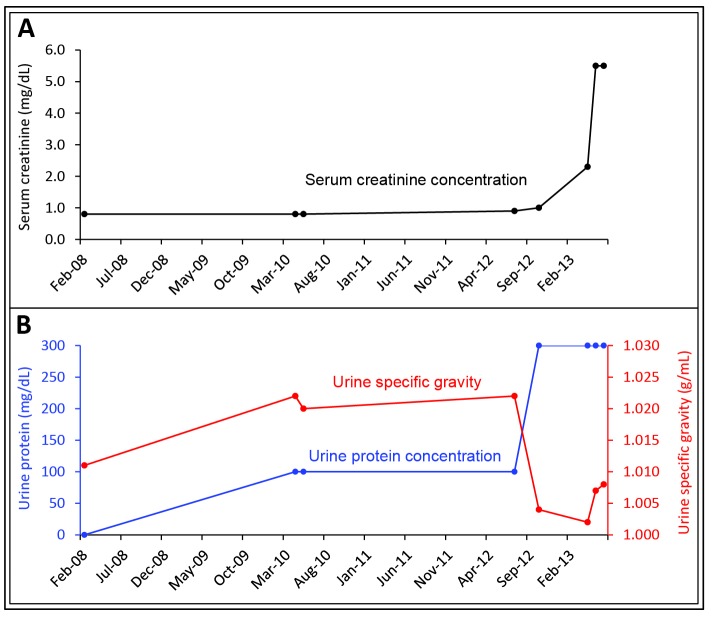
Renal function over time. A rise in serum creatinine concentration (A) was accompanied by a fall in urine specific gravity and an increase in urinary protein excretion (B).

Renal AA amyloidosis was believed to be secondary to chronic suppurative skin infections complicating subcutaneous drug injection. Acute tubular necrosis was probably secondary to infections and cocaine use. The patient received supportive care for renal disease and did not require renal replacement therapy. He received counseling for drug addiction and is currently awaiting aortic valve replacement.

## Discussion

We present a middle-aged man with a 5-year history of intravenous and subcutaneous drug use resulting in recurrent skin abscesses. He developed isosthenuria, proteinuria, and progressive renal insufficiency. Renal biopsy demonstrated AA amyloidosis.

Amyloidoses are a heterogeneous group of disorders characterized by the extracellular deposition of insoluble abnormal proteins in a β-pleated sheet configuration with distinctive tinctorial properties (1). AA amyloidosis is a systemic form of amyloidosis that is a complication of chronic infections and inflammatory disorders. AA amyloid is derived from serum amyloid A (SAA), which is an acute-phase protein released from the liver. SAA concentrations usually parallel those of CRP and can be increased as much as 1000-fold during an acute-phase response (2). Under physiological conditions, SAA is taken up by macrophages and degrades in the lysosomes (3). In patients with AA amyloidosis, however, partially cleaved SAA accumulated in the extracellular space, aggregate to form fibrils and associate with several glycosaminoglycans, lipids and serum amyloid P (SAP) (3,4). The final product, AA amyloid, is resistant to proteolysis. Only a subset of patients with chronic inflammatory disorders and elevated SAA concentrations develop amyloidosis (1,3). The type and duration of inflammatory disorders, SAA concentrations, and SAA polymorphisms are risk factors for the development of AA amyloidosis.

AA amyloidosis can be a complication of recurrent suppurative skin infections secondary to subcutaneous administration of narcotics (5,6). In most cases, drugs are injected subcutaneously due to unavailability of intravenous access sites. In a prospective study, seven cases of renal AA amyloidosis were found among 150 drug addicts examined at autopsy (5). Renal AA amyloidosis occurred in six of 44 (14%) subcutaneous users, but in only one of 105 (1%) intravenous users. Extensive skin infections were present in six of seven drug addicts with renal AA amyloidosis. Six of 23 (26%) drug addicts with skin infections had renal AA amyloidosis. The authors concluded that drug addicts who are subcutaneous users with skin infections are at highest risk for developing renal AA amyloidosis. In the study by Neugarten *et al*, the average duration of intravenous and subcutaneous drug abuse was 16 and 3 years, respectively (6). In line with these observations, our patient developed renal AA amyloidosis as a result of chronic skin abscesses secondary to subcutaneous drug injection.

Renal disease is a common and serious complication of AA amyloidosis (5-7). Accumulation of AA amyloid in the kidney results in proteinuria and progressive renal disease. Hypoalbuminemia secondary to proteinuria and sustained inflammation leads to edema. Tubular dysfunction can cause nephrogenic diabetes insipidus and renal tubular acidosis. In advanced cases, renal ultrasonography may demonstrate enlarged kidneys. In the study by Neugarten *et al*, the average serum creatinine concentration was 2.5 mg/dL, and urinary protein excretion averaged 18.8 g/day (6). In the study by Lachmann *et al*., renal dysfunction was present in the vast majority of the patients (7). Forty-one patients (11%) had end-stage renal disease. At the time of enrollment, median serum creatinine concentration and urinary protein excretion in those not requiring dialysis were 1.2 mg/dL and 3.9 g/day, respectively. Our patient developed the nephrotic syndrome characterized by heavy proteinuria, hypoalbuminemia, hypercholesterolemia, and edema. At the same time, he developed isosthenuria likely secondary to nephrogenic diabetes insipidus. Before the onset of acute tubular necrosis as a result of cutaneous and intravascular infections as well as cocaine use, he developed progressive renal insufficiency. The estimated glomerular filtration rate (CKD-EPI) declined 7.6 ml/min/1.73 m^2^ per year over the preceding 30 months.

The natural history and outcome of AA amyloidosis was evaluated in a study involving 374 patients followed for a median of 86 months (7). Most patients (82%) were White. The median age at diagnosis was 50 years. The more common underlying disorders were chronic inflammatory arthritis (60%), chronic infections (15%), periodic fever syndromes (9%) and Crohn’s disease (5%). Chronic infections were secondary to injection-drug use in 23% of patients. The average duration of the underlying inflammatory disorder was 17 years. Median SAA and CRP concentrations were 28 mg/L and 20 mg/L, respectively. The median survival from diagnosis was approximately 11 years. The SSA concentration correlated directly with amyloid burden estimated using whole-body ^123^I-SAP scintigraphy. In addition, the SAA concentration significantly correlated with the risk of death and progression of renal disease. Patients with chronic infections as the cause of AA amyloidosis had a worse renal outcome. Although we failed to measure SAA concentrations, CRP concentrations measured at three different time points were elevated at 7.2 mg/dL, 16.6 mg/dL, and 2.3 mg/dL (normal: <1.0 mg/dL). As stated above, CRP concentrations usually parallel those of SAA.

Although renal disease in AA amyloidosis confers a poor prognosis, deposition of AA amyloid also occurs in the spleen, adrenal glands and gastrointestinal tract (7). To determine the distribution and burden of amyloid, SAP scintigraphy can be performed (7,8). SAP scintigraphy demonstrated AA amyloid deposition in the spleen, adrenal glands and liver in 100%, 41% and 23% of patients, respectively (7). In 89% of patient, SAP scintigraphy demonstrated involvement of the kidneys, adrenal deposits or both. Although we did not perform SAP scintigraphy, our patient demonstrated AA amyloid deposition in the kidneys and likely in the liver and spleen (hepatomegaly noted on ultrasonography).

The diagnosis of AA amyloidosis is frequently overlooked due to the insidious nature of the disease. Renal AA amyloidosis should be suspected in patients suffering from a chronic inflammatory disease who present with proteinuria. Polarizing microscopy demonstrates characteristic apple-green birefringence of amyloid when stained with Congo red. Ultrastructural examination demonstrates 7-10 nm fibrils haphazardly distributed in the extracellular space.

Successful treatment of the underlying inflammatory disorders reduces SAA concentrations and arrests new AA amyloid formation and deposition (9-11). This approach improves survival and results in regression of amyloid deposits in a significant number of patients. Colchicine prevents AA amyloidosis in high-risk patients with familial Mediterranean fever and arrest the progression of renal disease in a subset of patients (12). In addition, colchicine was used with good results in a patient with AA amyloidosis due to drug abuse (13). Eprodisate is a compound that interferes with interactions between amyloid precursor proteins and glycosaminoglycans, thereby inhibiting polymerization and deposition of amyloid fibrils (14). Although it prevents new amyloid formation, eprodisate has no effect on the concentration of SAA and might not reduce the formation of SAA oligomers or protofibrils. In mouse models of AA amyloidosis, eprodisate was shown to inhibit amyloid deposition in tissue. Therefore, the efficacy and safety of eprodisate in the treatment of AA amyloidosis with kidney involvement was evaluated in a randomized, double-blind, placebo-controlled trial in humans (15). Patients (n = 183) received either eprodisate or placebo for 24 months. The creatinine clearance declined 10.9 and 15.6 ml/min/1.73 m^2^/year in the eprodisate and placebo groups, respectively. Although eprodisate was shown to slow down the decline in renal function in AA amyloidosis, there was no significant effect on proteinuria, progression to ESRD, and overall mortality. The incidence of adverse events was similar in the two groups.

## Conclusion

We presented a middle-aged drug addict who developed renal AA amyloidosis as a result of chronic cutaneous infections secondary to skin-popping. Although he showed early signs of renal disease, i.e. proteinuria and isosthenuria, for years, the diagnosis of renal AA-amyloidosis was established very late during the course of the disease. Accumulating evidence indicates that normalization of circulating SAA concentrations arrest AA amyloid deposition and is the primary objective in the treatment of AA amyloidosis. Furthermore, the institution of a treatment strategy should be accompanied by close monitoring of circulating SSA levels to ensure the effectiveness of the treatment. In a significant number of patients, normalization of circulating SAA concentrations is associated with reversal of organ damage as a result of regression of amyloid deposits. This report calls for a heightened awareness of AA amyloidosis as a potential cause of renal disease in patients with chronic inflammatory diseases such as recurrent bacterial skin infections due to subcutaneous drug use.

## Authors’ contributions

JMM, VP, MAP and AN wrote the manuscript. DBT made substantial contributions to the diagnosis and discussion.

## Conflict of interests

The authors have no conflicts of interest to declare.

## Ethical considerations

Ethical issues (including plagiarism, data fabrication and double publication) have been completely observed by the author.

## Funding/Support

This work received no funding from public, commercial or not-for-profit organizations.

## References

[1] Pepys MB (2006). Amyloidosis. Annu Rev Med.

[2] Gabay C, Kushner I (1999). Acute-phase proteins and other systemic responses to inflammation. N Engl J Med.

[3] Van der Hilst JC (2011). Recent insights into the pathogenesis of type AA amyloidosis. Scientific World Journal.

[4] Merlini G, Bellotti V (2003). Molecular mechanisms of amyloidosis. N Engl J Med.

[5] Menchel S, Cohen D, Gross E, Frangione B, Gallo G (1983). AA protein-related renal amyloidosis in drug addicts. Am J Pathol.

[6] Neugarten J, Gallo GR, Buxbaum J, Katz LA, Rubenstein J, Baldwin DS (1986). Amyloidosis in Subcutaneous Heroin Abusers (“skin popper’s amyloidosis). Am J Med.

[7] Lachmann HJ, Goodman HJ, Gilbertson JA, Gallimore JR, Sabin CA, Gillmore JD, Hawkins PN (2007). Natural history and outcome in systemic AA amyloidosis. N Engl J Med.

[8] Hawkins PN, Lavender JP, Pepys MB (1990). Evaluation of systemic amyloidosis by scintigraphy with 123I-labeled serum amyloid P component. N Engl J Med.

[9] Gillmore JD, Lovat LB, Persey MR, Pepys MB, Hawkins PN (2001). Amyloid load and clinical outcome in AA amyloidosis in relation to circulating concentration of serum amyloid A protein. Lancet.

[10] Nayer A (2014). Amyloid A amyloidosis: frequently neglected renal disease in injecting drug users. J Nephropathology.

[11] Cooper C, Bilbao JE, Said S, Alkhateeb H, Bizet J, Elfar A (2013). Serum amyloid A renal amyloidosis in a chronic subcutaneous (“skin popping”) heroin user. J Nephropathol.

[12] Zemer D, Pras M, Sohar E, Modan M, Cabili S, Gafni J (1986). Colchicine in the prevention and treatment of the amyloidosis of familial Mediterranean fever. N Engl J Med.

[13] Tan AU Jr, Cohen AH, Levine BS (1995). Renal amyloidosis in a drug abuser. J Am Soc Nephrol.

[14] Gervais F, Morissette C, Kong X (2003). Proteoglycans and amyloidogenic proteins in peripheral amyloidosis. Curr Med Chem Immun Endocr Metab Agents.

[15] Dember LM, Hawkins PN, Hazenberg BP, Gorevic PD, Merlini G, Butrimiene I (2007). Eprodisate for AA Amyloidosis Trial GroupEprodisate for the treatment of renal disease in AA amyloidosis. N Engl J Med.

